# Case of Ulcerated Leg, and Cases of Second Attack of Small-Pox

**Published:** 1806-03

**Authors:** C. Simpson

**Affiliations:** Skipton


### Case of Ulcerated Leg, and Cases of Second Attack of Small-Pox; *by* Mr. C. Simpson, *of Skipton*.

About eighteen months ago, I was requested to visit Mrs. P. of a very thin spare habit, who for upwards of thirty years had been much troubled with an inveterate ulcer, situated upon the external part of the right leg. The symptoms I found her labouring under, were as follow. A large, dark coloured ulcer, extending from the external ankle, nearly up to the calf of the leg, and almost round the tibial bone, the bottom of a sloughy appearance, discharging a large quantity of thin, acrid fluid, and offensive smell; the edges of the ulcer greatly thickened, and extremely irritable; the whole of the leg above the wound and the foot, very much enlarged and indurated; pulse 96, and small; constant thirst; restless nights, from severe pain of the leg; no appetite; great depression of spirits, and general debility. The plan she had always been advised to pursue, was a reducing one; strictly to avoid wine, malt liquor, animal food, &c. &c. and frequently to take brisk cathartics; being always taught to believe, if ever the ulcer was completely healed, the consequence would be fatal; under that idea, poultices and mild ointments formed the external treatment.

Finding her general health considerably impaired, the ulcer daily enlarging, with the aggravation of every other unpleasant symptom, she resolved to consult some other practitioner. From a full consideration of the case, I had no hesitation in reversing the treatment. I requested her to take the following powder three times a day, in a generous glass of red wine: ℞. Pulv. cinchon. flav. ℈j. pulv. aromaticæ, gr. v. M. ft. pulv.; also, one of the pills every night at bed time: ℞. Hydr. muriat. mitis, opii. pur. a. ℈j. ext. gentianæ. ʒj. M. ft. mass. in pil. No. xx. divid. together with a generous diet, the use of animal food, porter, &c. &c. the ulcer to be covered with a fresh carrot poultice, and renewed two or three times a day; the indurated parts above and below the wound, to be well rubbed twice a day, with the vol. camphorated liniment. As she resided eight miles from my place of abode, I did not see her again for six days, when I found the symptoms more favorable; her appetite improving; better nights; leg more easy; bark agreeing well; discharge from the ulcer less in quantity, and not offensive; pulse 84, of better strength; bowels regular; no effect upon the mouth from the mercury, cury. The bark to be continued, and increased to gr. xxv also the pill at bed-time. The carrot poultice was ordered to be omitted, and the wound dressed with lint soaked in the following balsam: ℞. Ung. res. flav. ℥iß. bals. canad. ʒß. M. twice a day, with a pledget of mild cerate over it; afterwards a flannel roller regularly applied, from the toes to the knee.

Six days afterwards I saw her again, and found her continuing convalescent. The bottom of the wound free from sloughs; healthy granulations shooting from the sides of the wound. The discharge of a good consistence; general strength recovering. The indurated parts softer, and the bulk of the limb considerably reduced. Her mouth affected from the mercury. Her bowels being rather costive, a mild laxative medicine was ordered; after its operation, the bark and pills to be continued, omitting the calomel; strict attention to be paid to the proper application of the roller. By pursuing this mode of treatment ten days longer, the granulations had so far increased, as to be nearly level with the surface. The balsamic ointment was then discontinued, and long strips of fine linen spread with the cerat. lap. calam. (of a firm consistence) was applied round the leg, tight enough to contract the edges of the wound moderately; compresses of lint were laid over them, and the roller as before. With this treatment, cicatrization commenced, and the wound was entirely healed in three weeks. She has ever since enjoyed a better state of health than she had for upwards of thirty years.

Had my patient continued the plan I found her pursuing, much longer, I am confident the discharge from the wound would soon have exhausted her.

I have frequently found in old ulcerated legs, a free stimulus given to the absorbent system attended with the very best effects. In constitutions that have been injured by the too free use of spirits, I have often found the application of the common adhesive straps excite so much irritation, as to oblige me to discontinue them; but by making use of the cerat. lap. calam. spread upon strips of fine linen, and an opiate twice a day for the space of ten days, I have then been able to use them as in other cases.

---

I beg leave to acquaint Mr. Ring, through the medium of your Journal, with the following facts, relative to the second attack of small-pox, in particular idiosyncracies.

A few

A few days ago, I called upon a Mrs. Wilkinson of this place, who, I was informed had a son, that had had the small-pox after inoculation. She told me, when he was a few months old, he was inoculated for the small-pox by a Mr. Hall (formerly a respectable practitioner in this place, since dead) that he had the complaint in a very severe manner, with a great number of very fine pustules, and a great degree of fever for several days. That Mr H. expressed his entire satisfaction, with respect to his security from a future attack. Seven years afterwards, the small-pox raging here, he caught the infection from some neighbouring children, sickened, had a very full crop of pustules, which to this date bear proof of the severity of the attack, and from which he recovered with great difficulty.

She further told me, she had a brother who had the *natural small-pox twice.* As he resides in this place, I immediately waited upon him; he confirmed what his sister had told me, by saying he perfectly remembered both attacks, being twelve years old when he had the first, and fourteen the second; the first was a very severe one, the second though not quite so severe, yet by no means a mild disease. Several children were inoculated from the pus tules in both attacks.

